# The formation, phase behavior and properties of helium fluoroperovskites as seen by in-situ X-ray and neutron powder diffraction

**DOI:** 10.1063/4.0000847

**Published:** 2025-10-27

**Authors:** Angus P Wilkinson, Shangye Ma, Jamie Molaison, Antonio dos Santos

**Affiliations:** 1 Georgia Institute of Technology, Atlanta, GA, USA; 2 Oak Ridge National Laboratory, Oak Ridge, TN, USA

## Abstract

Fluoroperovskites with helium on their A-sites can be prepared by compressing the ReO3-type materials CaZrF6, CaNbF6 and ScF3 in helium at temperatures above ∼150 K. The insertion of helium to form [He2-x]CaZrF6, [He2-x]CaNbF6 and [Hex]ScF3 in some cases dramatically changes the phase behavior and properties of the parent fluoride. Notably, helium incorporation reduces the magnitude of the negative thermal expansion seen in the parent phase, and stiffens the structure. Also, on compressing CaZrF6 in a non-penetrating fluid, an amorphization is observed at 0.5 GPa, but in helium the crystalline perovskite He2CaZrF6 persists to ∼3.5 GPa. In the case of ScF3, compression in a non-penetrating fluid leads to a cubic to rhombohedral phase transition at ∼0.7 GPa and 300 K, and at < 0.1 GPa for 10 K. However, the incorporation of small amounts of helium, to form He0.1ScF3, raises the cubic to rhombohedral phase transition pressure to ∼0.2 GPa at 10 K, and on compressing ScF3 in helium at 300 K, the cubic to rhombohedral transition is not observed, but other structural phase transitions for HeScF3 are seen. High pressure neutron diffraction is a particularly valuable probe of perovskite formation and behavior, as it enables the crystallographic quantification of helium in the perovskites.

